# On the role of spatial phase and phase correlation in vision, illusion, and cognition

**DOI:** 10.3389/fncom.2015.00045

**Published:** 2015-04-21

**Authors:** Evgeny Gladilin, Roland Eils

**Affiliations:** ^1^Division of Theoretical Bioinformatics, German Cancer Research CenterHeidelberg, Germany; ^2^BioQuant and IPMB, University HeidelbergHeidelberg, Germany

**Keywords:** vision research, visual illusions, motion detection, pattern recognition, saccades, acuity, phase correlation, association cortex

## Abstract

Numerous findings indicate that spatial phase bears an important cognitive information. Distortion of phase affects topology of edge structures and makes images unrecognizable. In turn, appropriately phase-structured patterns give rise to various illusions of virtual image content and apparent motion. Despite a large body of phenomenological evidence not much is known yet about the role of phase information in neural mechanisms of visual perception and cognition. Here, we are concerned with analysis of the role of spatial phase in computational and biological vision, emergence of visual illusions and pattern recognition. We hypothesize that fundamental importance of phase information for invariant retrieval of structural image features and motion detection promoted development of phase-based mechanisms of neural image processing in course of evolution of biological vision. Using an extension of Fourier phase correlation technique, we show that the core functions of visual system such as motion detection and pattern recognition can be facilitated by the same basic mechanism. Our analysis suggests that emergence of visual illusions can be attributed to presence of coherently phase-shifted repetitive patterns as well as the effects of acuity compensation by saccadic eye movements. We speculate that biological vision relies on perceptual mechanisms effectively similar to phase correlation, and predict neural features of visual pattern (dis)similarity that can be used for experimental validation of our hypothesis of “cognition by phase correlation.”

## 1. Introduction

Continuous evolution of biological systems implicates a common origin of different functions and mechanisms that emerged as a result of successive modification of one particularly advantageous basic principle. Electrophysiological findings (Hubel and Wiesel, 1968) and psychophysical experiments (Campbell and Robson, 1968) indicate that visual system relies on the basic principle of frequency domain transformation of the retinal image in visual cortex which was initially believed to resemble a crude Fourier transformation (Graham, 1981). Even though, more recent mathematical models of sparse image coding revised the assumption of global Fourier transformation in favor of locally supported Gabor- (Marcelja, 1980), Wavelet- Mallat, 1989, Wedge-, Ridge- or Curvelet-functions (Donoho and Flesia, 2001), the concept of neural image representation in the frequency domain by phase and amplitude remained valid.

Since pioneering works of Hubel and Wiesel (1962, 1968), Campbell and Robson (1968), Blakemore and Campbell (1969), Blakemore et al. (1969), and Thomas et al. (1969) it is known that different groups of neurons in the visual cortex show selective response to spatial-temporal characteristics of visual stimuli and operate as spatially organized filters (receptive fields) that extract particular image features (i.e., spatial frequency, orientation) within a certain range (bandwidth) of their sensitivity. Numerous subsequent studies dealt with experimental investigation and theoretical modeling of visual receptive fields and analysis of their amplitude-transfer (ATF) and phase-transfer functions (PTF). The existing body of evidence resulting from four decades of research on this field includes

- existence of frequency-selective V1 neurons operating as bandpass filters (Graham, 1989; De Valois and De Valois, 1990),
- coding of phase information using quadrature pairs of bandpass filters (Pollen and Ronner, 1983),
- odd-/even-symmetric filters in visual cortex (Morrone and Owens, 1987),
- linear ATF and PTF of simple striatic neurons (Hamilton et al., 1989),
- computation of complex-valued products in V1 neurons (Ohzawa et al., 1990),
- computation of magnitudes (energies) in complex V1 cells as a sum of squared responses of simple V1 cells (Adelson and Bergen, 1985),
- divisive normalization of neuronal filter responses (Heeger, 1992; Schwartz and Simoncelli, 2001),
- motion detection (Fleet and Jepson, 1990; Nishida, 2011),
- edge detection (Kovesi, 2000; Henriksson et al., 2009),
- stereoscopic vision (Fleet, 1994; Fleet et al., 1996; Ohzawa et al., 1997),
- 3D shape perception (Thaler et al., 2007),
- assessment of pattern similarity (Sampat et al., 2009; Zhang et al., 2014),
- triggering of diverse visual illusions (Popple and Levi, 2000; Backus and Oru, 2005).

Altogether, these findings support the concept of neural transformation of retinal images into frequency domain characteristics (i.e., phase and amplitude) that, in turn, serve as an input for subsequent higher-order mechanisms and functions of visual perception and cognition.

Despite recent advances in understanding of the overall topology and hierarchy of visual cortex (Riesenhuber, 2005; Poggio and Ullman, 2013), little is known yet about the underlying wiring schemes of phase/amplitude information processing in visual cortex. In particular, the observation that small cells of V1 show phase-sensitivity (Pollen and Ronner, 1981) while complex cells do not (De Valois et al., 1982) lead to controversial discussion about the role of spatial phase in visual information processing (Morgan et al., 1991; Bex and Makous, 2002; Shams and Malsburg, 2002; Hietanen et al., 2013).

In what follows we aim to address the following basic questions:

- What are the driving forces behind the evolutionary development of biological vision?
- What properties of spatial phase (further in this manuscript denoted as phase) make it an important feature for visual information processing?
- What is the origin of various phase-related visual phenomena including illusions of apparent motion, stereograms and virtual image context?
- How can phase information be used for motion detection and (dis)similarity cognition, and how can theoretical models be evaluated experimentally?

Our manuscript is organized as follows. First, we recapitulate the role of environmental constraints in development of biological vision in course of evolution. We review theoretical properties of phase using an extension of the Fourier phase correlation technique and demonstrate how phase information can be used for edge enhancement, motion detection, and pattern recognition. We show that saccadic strategy of image sampling naturally emerges within this concept as an algorithmic solution which improves the confidence of visual pattern discrimination and recognition. Further, we apply the concept of phase shift and correlation to analysis of different visual illusions and hypothesize about involvement of phase-based mechanisms in perception of motion and visual pattern (dis)similarity. In conclusion, we make suggestions for experimental evaluation of our theoretical predictions.

## 2. Invariants of ecological environment and evolution of vision

**Figure 1:**
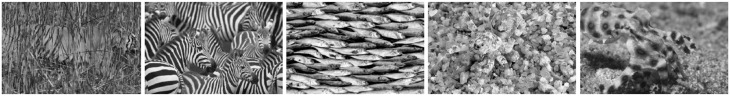
**Repetitive patterns, swarm motion, and body morphing disrupt detection of unique invariant features (i.e., rigid animal silhouettes)**. Examples of natural images are acquired from public Creative Commons sources (http://search.creativecommons.org/).

The evolutionary principle implies that remarkable abilities of biological vision result from adaptation of species to the environmental constraints that ancestors had to cope with in the past. It is generally recognized that progressive sophistication of vision is driven toward more efficient representation, processing and, probably, also modeling of the physical reality which stands behind the retinal images (Walls, 1962; Marr, 1982; Hyvärinen and Hoyer, 2001; Graham and Field, 2006). In addition to the basic optosensory function, the core tasks of visual perception in macroscopic organisms include orientation in the physical environment, which premises ability to detect obstacles and relative motion, as well as recognition of essential patterns related to food, threat and communication. Further, we recollect that biological organisms are composed of condensed matter and have to mainly take care about the objects of the physical world that also have rigid constitution and conservative shape. In contrast, highly deformable media such as gasses and liquids are biologically neutral which implicates that perception of non-rigid transformations did not fall under the early evolutionary pressure. Important is the notion that visual perception of rigid bodies with a preserved shape has to be independent on relative spatial position and orientation which means that it has to rely on some invariants (Ito et al., 1995; Booth and Rolls, 1998; Palmeri and Gauthier, 2004; Lindeberg, 2013) that are not given *per se* but have to be derived by subsequent processing of the raw retinal image. As a dimensionless quantity, phase bears topological information independently on the level of illuminance and contrast. Affine transformations in the image domain do not change the relative phase structure, but merely shift it as a whole. These properties of phase are of advantage for survival of the fittest and can be assumed to be “discovered” in course of the evolution of biological vision. Different features of visual perception emerge at evolutionarily distant time points and, thus, rely on different intrinsic invariances. Early forms of life are originated in the marine environment where movements are slowed down by viscosity of water, effects of gravitation are diminished and changes in the relative spatial position and orientation are more probable as it is the case in terrestrial environment with its stable gravitational axis and unresisting atmosphere. The ability to recognize abstract shapes (i.e., animal silhouettes) independently on their relative motion, orientation, and distance was essential to survival of species and probably originated already with the first marine animals. However, the translation-, rotation-, scaling-independent (i.e., TRS-invariant) perception of abstract shapes (Gladilin, 2004) does not apply to all kinds of visual stimuli. A prominent example of dependency of visual perception on changing environmental constraints is the Thatcher-Illusion, which consists in poor recognition of upside-down faces (Psalta et al., 2014). Comparative experiments with different primates demonstrate that perception of facial expression is a relatively new feature in biological vision (Weldon et al., 2013). Sensitivity of human face perception to rotations has obviously to do with the fact that the neuronal machinery of face recognition is relatively new cognitive feature which emerged in the terrestrial environment where primates encountered each other predominantly in the upright posture. In general, visual illusions can be attributed to optical stimuli that mislead evolutionarily conserved mechanisms of visual information processing based on a built-in knowledge of properties of the physical world (Ramachandran and Anstis, 1986). The ability to irritate or escape common cognitive schemes is, in turn, of evolutionary advantage. The fact that many animals use camouflage patterning, swarm motion or body morphing as a reliable survival strategy indicates that repetitive patterns and non-TRS transformations represent a principle challenge for biological vision which is evolutionarily predetermined to rely on TRS-invariants of the condensed matter world, see Figure 1.

## 3. The role of phase from the viewpoint of computer vision

In this section, we elucidate the role of phase information for detection of image motion and pattern recognition from the viewpoint of computer vision. Readers who are not familiar with Fourier analysis may skip over math-intensive parts that will be concluded subsequently.

### 3.1. Image representation in spatial and frequency domains

In spatial domain, 2D images are represented by a matrix *A*_*x*, *y*_ of *N* × *M* scalar intensity values on an Euclidian image raster (*x* ∈ [0, *N* − 1], *y* ∈ [0, *M* − 1]). Complex Fourier transformation maps an image *A*_*x*, *y*_ onto the complex frequency domain α_*u*, *v*_: 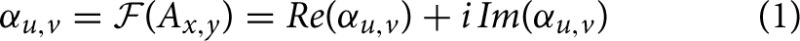 or in a more explicit form for a discrete 2D case:

(2) $$\alpha_{u,v}=\frac{1}{\sqrt{MN}}\sum_{x\;=\;0}^{N\;-\;1}\sum_{y\;=\;0}^{M\;-\;1}A_{x,y}e^{-2\pi i\left(\frac{ux}{N}+\frac{vy}{M}\right)}.$$

The inverse Fourier transformation mapping α_*u*, *v*_ onto the spatial domain is given by 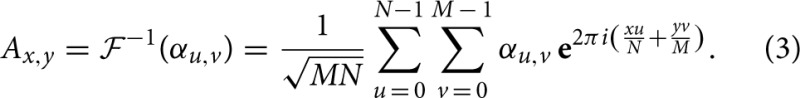


Further, we recollect that the complex conjugate of α_*u*, *v*_ is defined as α^*^_u, *v*_ = *Re*(α_*u*, *v*_) − *i Im*(α_*u*, *v*_).

### 3.2. Importance of phase and amplitude: theoretical perspective

**Figure 2:**
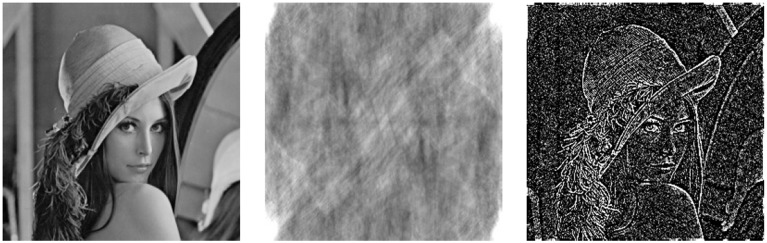
**Comparison of the effects of amplitude and phase distortion on image reconstruction**. From left to right: the original Lenna image vs. amplitude-only and phase-only image transforms. The phase-only transformation works as an edge-enhancing filter resembling the Marr's Primal Sketch (Marr, 1982).

The relative importance of Fourier phase and amplitude for retrieval of structural image features has been debated in several previous works (Oppenheim and Lim, 1981; Lohmann et al., 1997; Ni and Huo, 2007). The basic notion is that the phase bears topological information about image edges whereas amplitude encodes image intensity. To demonstrate the effect of amplitude and phase distortion, we perform reconstruction of the original image from amplitude-only and phase-only of its Fourier transform, see Figure 2. Here, the amplitude-only reconstruction (Figure 2 (middle)) is computed as the Fourier inverse of the following amplitude-preserving and phase-eliminating transformation:

(4) $$\begin{array}{l} Re(\alpha_{u,v})\to {\left(Re{\left(\alpha_{u,v}\right)}^{2}+Im{\left(\alpha_{u,v}\right)}^{2}\right)}^{1/2},\\ Im(\alpha_{u,v})\to 0, \end{array}$$

and the phase-only reconstruction (Figure 2 (right)) is calculated as the Fourier inverse of the following phase-preserving and amplitude-normalizing transformation:

(5) $$\begin{array}{l} Re(\alpha_{u,v})\to \frac{Re(\alpha_{u,v})}{{\left(Re{\left(\alpha_{u,v}\right)}^{2}+Im{\left(\alpha_{u,v}\right)}^{2}\right)}^{1/2}},\\ Im(\alpha_{u,v})\to \frac{Im(\alpha_{u,v})}{{\left(Re{\left(\alpha_{u,v}\right)}^{2}+Im{\left(\alpha_{u,v}\right)}^{2}\right)}^{1/2}}. \end{array}$$

This example demonstrates that the relative phase appears to be more significant for retrieval of cognitive image features (i.e., edges) that get completely lost in the amplitude-only transformation. Remarkably, the amplitude-normalizing phase-only reconstruction seem to effectively work as an edge-enhancing filter which generates a feature-preserving image sketch resembling the Marr's concept of the Primal Sketch generation in visual cortex (Marr, 1982).

### 3.3. Detection of uniform image motion using phase correlation

The Fourier phase correlation (*PC*) is a powerful technique which has been originally developed for detection of affine image transformations such as uniform translational motion, rotation and/or scaling (De Castro and Morandi, 1987; Reddy and Chatterji, 1996). Phase correlation between two images *A*_*x*, *y*_ and *B*_*x*, *y*_, is computed as a Fourier inverse of the normalized cross-power spectrum (*CPS*): 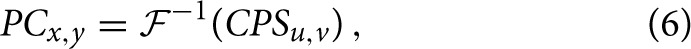 where

(7) $$CPS_{u,v}=\frac{\alpha_{u,v}\beta_{u,v}^{*}}{\left|\alpha_{u,v}\beta_{u,v}^{*}\right|}$$

and 
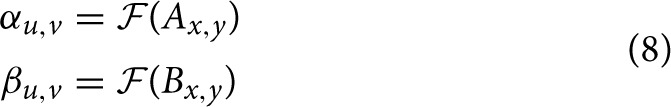
 are the complex Fourier transforms of the images *A*_*x*, *y*_ and *B*_*x*, *y*_, respectively. According to the Fourier shift theorem, relative displacement (Δ*x*, Δ*y*) between two identical images, i.e.,

(9) $$B_{x,y}=A_{x-\Delta x,y-\Delta y},$$

corresponds to phase-shift in the frequency domain

(10) $$\beta_{u,v}=e^{-2\pi i\varphi }\alpha_{u,v},$$

where $\varphi =(\frac{u\Delta x}{N}+\frac{v\Delta y}{N})$. Consequently, the cross power spectrum between two identical images shifted with respect to each other in the spatial domain describes the phase-shifts of the entire Fourier spectrum in the frequency domain:

(11) $$CPS_{u,v}=\frac{\alpha_{u,v}e^{2\pi i\varphi }\alpha_{u,v}^{*}}{\left|\alpha_{u,v}e^{2\pi i\varphi }\alpha_{u,v}^{*}\right|}=e^{2\pi i\varphi }.$$

For two identical images with the relative spatial shift (Δ*x*, Δ*y*), the inverse Fourier integral of Equation (11), i.e., the phase correlation Equation (6), exhibits a single singularity at the point (*x* = Δ*x*, *y* = Δ*y*) and is given by

(12) $$PC_{x,y}=\delta (x-\Delta x,y-\Delta y).$$

**Figure 3:**
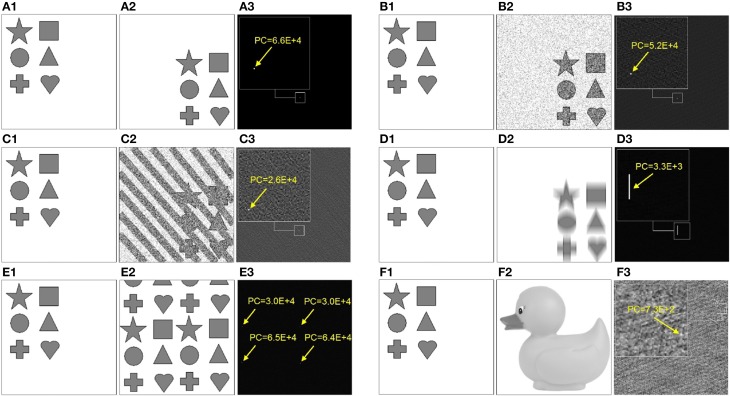
**Examples of phase correlation (right column) between the source (left column) and the target image (middle column)**. Target images **(A2-E.2)** represent the following transformations of the source image: **(A2)** uniform displacement, **(B2)** uniform displacement superimposed with 70% statistical noise, **(C2)** uniform displacement superimposed with 70% statistical and structural noise, **(D2)** uniform displacement superimposed with 20-pixel Y-motion-blur, **(E2)** superposition of four different uniform displacements (i.e., 4× fold repetition). **(F)** shows phase correlation between two significantly different images. Arrows point to the location of the absolute maximum peak of the *PC*. Visualization of the entire *PC* is performed using the following grayscale mapping: *PC*_*x*, *y*_ → 255(*PC*_*x*, *y*_ − MIN(*PC*_*x*, *y*_))/(MAX(*PC*_*x*, *y*_) − MIN(*PC*_*x*, *y*_)).

Thus, phase correlation of two identical images has a single maximum-peak which coordinates in the spatial domain yield the relative image translation^1^ (*x* = Δ*x*, *y* = Δ*y*), see Figure 3A.

### 3.4. Phase correlation in the presence of noise

In the presence of additive statistical or structural noise, the cross power spectrum between two non-identical images takes the form:

(13) $$CPS_{u,v}=e^{2\pi i\varphi }+\varepsilon_{u,v},$$

where ε_*u*, *v*_ is a frequency-dependent perturbation-term whose properties depend on particular type of image differences. Consequently, the inverse Fourier integral of Equation (13), i.e., the phase correlation between two non-identical images, becomes different from the Dirac delta peak of the identical image shift Equation (12): 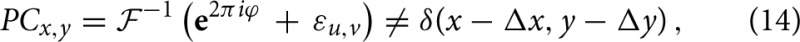 which manifests in flattening of the maximum peak and overall more noisy *PC*, see Figures 3B,C. However, as long as the target pattern do not exhibit similarities with the background structures, phase correlation between two images remains a single-peak distribution. Remarkably, even a significant structural distortion does not affect the detection of the target pattern within the noisy visual scene, see Figure 3C. This example demonstrates that the height of maxima and the overall shape of the *PC* distribution can serve as quantitative characteristics of image (dis)similarity, i.e., the more sharp (Dirac-like) is the *PC* distribution, the more similar are the structures in the underlying images. An increasingly dispersed *PC* distribution indicates lower image similarity.

In the case of non-affine image transformations, phase correlation loses its exceptional properties and becomes a multi-peak distribution. Figure 3D shows the phase correlation of the original image with its blurred and displaced copy. Uncertainty of the 20-pixel Y-motion-blur applied in this example reflects in the horizontal line of peaks in *PC* that correspond to possible alignments between the original image with its transformed copy.

If the target pattern is multi-present or exhibits structural similarity with the surrounding structures, multiple peaks occur in *PC*. Figure 3E shows phase correlation between the target pattern and the image containing its four displaced copies. Finding the right correspondence in such visual scene becomes difficult or impossible. Camouflage textures and behavioral strategies of swarm animals generate repetitive patterns that irritate cognitive mechanisms of predators based on detection of unique target features, see Figure 1.

With increasing structural differences between each two images, *PC* becomes a random distribution with the significantly lower maximum peaks, see Figure 3F.

### 3.5. Phase correlation in the case of non-uniform image motion

Non-uniform motion means that displacements of image pixels differ in directions and/or magnitude. Consider time-series of images *A*_*x*, *y*_(*t*) that are composed of two non-uniformly moving regions:

(15) $$A_{x,y}(t)=P_{x,y}(t)+B_{x,y}(t),$$

where *P*_*x*, *y*_ stands for a particular image pattern which has to be tracked in consecutive time steps, and *B*_*x*, *y*_ is the background region. Let *P*_*x*, *y*_ and *B*_*x*, *y*_ in the subsequent time step *A*_*x*, *y*_(*t* + 1) undergo different translations:

(16) $$A_{x,y}(t+1)=P_{x,y}(t+1)+B_{x,y}(t+1),$$

where

(17) $$\begin{array}{l} P_{x,y}(t+1)=P_{x+\Delta x_{p},y+\Delta y_{p}}(t),\\ B_{x,y}(t+1)=B_{x+\Delta x_{b},y+\Delta y_{b}}(t). \end{array}$$

Considering the linearity of Fourier transformation, one obtains for 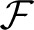
(*A*_*x*, *y*_(*t*)) and 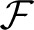
(*A*_*x*, *y*_(*t* + 1))

(18) $$\begin{array}{l} \alpha_{u,v}(t)=\rho_{u,v}+\beta_{u,v}\\ \alpha_{u,v}(t+1)=e^{-2\pi i\varphi }\rho_{u,v}+e^{-2\pi i\psi }\beta_{u,v}, \end{array}$$

where $\varphi =(\frac{u\Delta x_{p}}{N}+\frac{v\Delta y_{p}}{N})$ and $\psi =(\frac{u\Delta x_{b}}{N}+\frac{v\Delta y_{b}}{N})$, respectively. Consequently, the cross power spectrum between *A*_*x*, *y*_(*t*) and *A*_*x*, *y*_(*t* + 1) takes the form

(19) $$\begin{array}{l} CPS_{u,v}=\frac{\alpha_{u,v}(t)\alpha_{u,v}^{*}(t+1)}{\left|\alpha_{u,v}(t)\alpha_{u,v}^{*}(t+1)\right|}=\frac{1}{\left|\alpha_{u,v}(t)\alpha_{u,v}^{*}(t+1)\right|}\\ \;(\rho_{u,v}e^{2\pi i\varphi }\rho_{u,v}^{*}+\rho_{u,v}e^{2\pi i\psi }\beta_{u,v}^{*}+\\ \;\beta_{u,v}e^{2\pi i\varphi }\rho_{u,v}^{*}+\beta_{u,v}e^{2\pi i\psi }\beta_{u,v}^{*}) \end{array}$$

or in a more compact form

(20) $$CPS=CPS_{p^{\prime }}^{p}+CPS_{b^{\prime }}^{p}+CPS_{p^{\prime }}^{b}+CPS_{b^{\prime }}^{b},$$

where CPS^*^_*_ denote self- and cross-correlations between the Fourier transforms of the pattern and background regions in two consecutive time steps, respectively. Primed indexes are introduced to distinguish Fourier transforms of previous (*t*: *p*, *b*) and subsequent (*t* + 1: *p*′, *b*′) time steps. By applying the inverse Fourier transformation to Equation (20), one obtains the phase correlation between *A*(*t*) and *A*(*t* + 1): 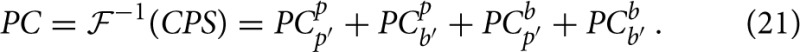


### 3.6. Saccades-enhanced phase correlation

Phase correlation between two non-uniformly shifted image regions Equation (21) contains four terms:

- self-correlation of the target pattern (*PC*^*p*^_*p*′_),
- self-correlation of the background region (*PC*^*b*^_*b*′_) and
- two cross-correlation terms (*PC*^*p*^_*b*′_, *PC*^*b*^_*p*′_).

**Figure 4:**
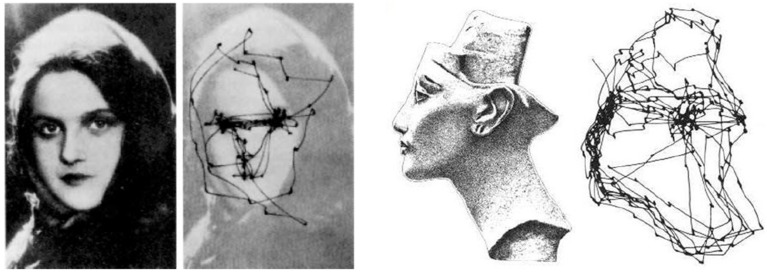
**Examples of saccadic eye movements from Yarbus (1967)**. **Left** the eyes of the observer exhibit remarkable back-and-forth movements between different regions of interest (i.e., eyes, mouth) and the image background. **Right** saccadic trajectories seem to follow the shape contours and edges.

In order to detect the shift of the target pattern *P*, *PC*^*p*^_*p*′_ has to become the most dominant term of the total *PC*. Obviously, this condition is not automatically fulfilled,—other terms may have stronger weight in Equation (21). If the pattern and background regions do not exhibit similarities, i.e., if the pattern *P* is uniquely present in the image, cross-correlation terms (*PC*^*p*^_*b*′_ and *PC*^*b*^_*p*′_) should be smaller in comparison to self-correlation terms (*PC*^*p*^_*p*′_ and *PC*^*b*^_*b*′_). Thus, the major difficulty for detection of the target image pattern is caused by self-correlation of the background region (*PC*^*b*^_*b*′_) which properties are *a priori* unknown. Obviously, a single-step phase correlation between two images is not sufficient for detection of a particular image region. In order to maximize the weight of *PC*^*p*^_*p*′_ and, correspondingly, to minimize the weight of other terms in Equation (21), one can construct a cumulative phase correlation by iteratively composing *PC* between the (fixed) target pattern with differently shifted background. Due to formal similarity of such strategy with back-and-forth image sampling by saccadic eye movements (see Figure 4), we termed this procedure saccades-enhanced phase correlation (Gladilin and Eils, 2009). To show why this strategy appears to be promising, we write the average phase correlation of *N* recombinations between the target pattern and non-uniformly shifted background images:

(22) $$\bar{PC}=\frac{1}{N}\sum_{i\;=\;1}^{N}PC_{i}=PC_{p^{\prime }}^{p}+PC_{b^{\prime }}^{p}+\frac{1}{N}\sum_{i\;=\;1}^{N}PC_{p^{\prime }}^{b_{i}}+\frac{1}{N}\sum_{i\;=\;1}^{N}PC_{b^{\prime }}^{b_{i}}.$$

Since first two terms in Equation (22) are independent on background variations (*b*_*i*_), their absolute values remain unchanged. Further, it can be shown that the last two terms decrease with increasing *N*, and, thus, their weight in the average phase correlation can be arbitrarily decreased after sufficiently high number of saccadic iterations *N* > > 1. Without providing a precise proof, we can give the following plausible comment: for different shifts of the background region, positions of maxima in cumulative phase correlation differ as well. Consequently, the sum over different *b*_*i*_ remains bounded, and the average value of the last two terms in Equation (22) decreases as *N*^−1^, i.e., $\lim_{N\to \infty }\left(\frac{1}{N}\sum_{i=1}^{N}PC_{b^{\prime }}^{b_{i}}\right)\to 0$. As a result of saccadic image composition, self-correlation of the target pattern *PC*^*p*^_*p*′_ becomes the most dominant term and the shift of *P* can be determined from the coordinate of the absolute maximum of Equation (22).

The less structured is the target pattern and the more similar it is to the image background, the more difficult becomes the virtual separation of target and background regions using saccades-enhanced phase correlation. Consequently, analysis of poorly structured visual scenes requires more saccadic iterations for detection and recognition of the target pattern. Remarkably, experimental findings seem to confirm this theoretical prediction: the strategy of saccades by observation of unstructured textural images exhibits increasing frequency of target-background eye movements (He and Kowler, 1992).

### 3.7. Consideration of visual acuity

The foveal and peripheral areas of the retinal image are known to exhibit significant differences in acuity that have to be considered by construction of Fourier transforms and phase correlations of target and surrounding images. With approximately 3° of high-acuity foveal cone-projection (Osterberg, 1935), the observer's eye can sharply resolve only an area with the cross-section dimension of *D* ≈ 0.1 *L*, where *L* denotes the distance from observer to the focus plane. For a *L* = 50 cm far computer screen, it makes a *D* = 5 cm wide spot. The remaining peripheral area is progressively blurred with the distance from the focus. Consequently, a more natural representation of the retinal and higher-lever neural images is the composition of the central pattern surrounded by the low-pass smoothed periphery. For calculation of saccades-enhanced phase correlation this, in turn, means that not only the position of the focus but also spectral characteristics of the central and peripheral areas have to be appropriately filtered anew for each saccadic fixation image. Repetitive target-background sampling by saccades will, obviously, lead to enhancement of small details (i.e., high-frequent components) of more frequently focused regions and low-pass smoothing of less frequently sampled, peripheral areas. As a consequence, one can expect saccadic analysis to better discriminate images that show distinctive spectral differences between central and peripheral areas. Visual examination of images with similar spectral characteristics of pattern and background regions can be, in turn, associated with intensification of back-and-forth saccadic eye movements.

## 4. Psychophysical evidence of phase involvement in visual information processing

In this section, we review some psychophysical findings indicating the involvement of phase in visual information processing and analyze them from the perspective of theoretical concepts of phase-based motion and pattern detection.

### 4.1. Importance of phase and amplitude: psychophysical perspective

From theoretical considerations in Section 3.2, phase appears to be more essential for retrieval of structural information than amplitude. Psychophysical findings in Freeman and Simoncelli (2011) and Zhang et al. (2014) suggest, however, a combined phase-amplitude mechanism of pattern perception with higher weight of phase information near the fixation point and increasing importance of amplitude on the periphery of the visual field. On the other hand, one should consider that conscious fixations inhibit saccades which results in progressive low-pass blurring of peripheral image. Unconstrained image observation is always associated with saccadic eye movements that acquire high-frequency phase information from different image areas and, thus, substantially increase the real weight of phase information in image perception and (re)cognition.

### 4.2. On the role of phase and saccades in visual illusions

Seemingly different visual illusions have a common feature to be triggered by coherently phase-shifted repetitive patterns. Below we briefly review three groups of visual illusions^2^ that generate effects of (i) virtual depth (Tyler and Clarke, 1990), (ii) apparent motion (Kitaoka and Ashida, 2003), and (iii) non-local image tilt (Popple and Levi, 2000). Tight resemblance in stimulus configuration of different visual illusions has been supposed in previous works (Kitaoka, 2006). Though, a unified concept of underlying neural mechanisms that drive different perceptual illusions is still missing.

#### 4.2.1. Virtual depth illusions

**Figure 5:**
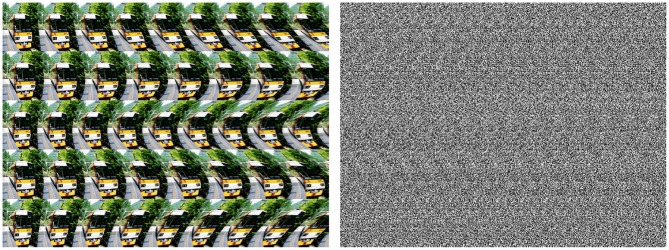
**Examples of virtual depth illusions (stereograms) based on structured (left) and diffuse textural (right) patterns (courtesy A. Kitaoka)**.

Stereogram images such as shown in Figure 5 cause perceptual illusions of virtual depth and hidden 3D content. Stereograms are composed of repetitive patterns which retinal projections in the left and right eyes exhibit a relative spatial shift in the image domain and a corresponding phase-shift in the frequency domain. Accordingly, two basic models of binocular disparity based on position- and phase-shift receptive fields have been discussed in the literature in the last two decades (Arndt et al., 1995; Fleet et al., 1996; Ohzawa et al., 1997; Parker and Cumming, 2001; Chen and Qian, 2004; Goutcher and Hibbard, 2014). Anzai et al. (1997) conclude that “binocular disparity is mainly encoded through phase disparity.” Fleet (1994) suggests a model of binocular disparity computation using the Local Weighted Phase Correlation which combines the features of phase-shift and phase correlation approaches. If phase correlation is, in fact, involved in binocular disparity calculation, the underlying neural mechanisms of virtual depth detection can be expected to depend on a certain threshold of neuronal activity, i.e., the strength of phase correlation, which, in turn, should be dependent on structural image properties. In particular, as we have seen above one can expect that structured (i.e., edge-rich, phase-congruent) patterns such as shown in Figure 5 (left) produce stronger phase correlation signals and, thus, trigger virtual depth illusions easier re. faster than diffuse textural pattern such as Figure 5 (right). Further experimental investigations are required to test this pure theoretical prediction.

#### 4.2.2. Apparent motion illusions

Apparent motion illusions induce perception of dynamic image changes while observing static visual stimuli. Notably, the intensity of apparent motion illusions depends on spectral characteristics (i.e., low/high frequent image content) and the relative phase-shift of repetitive patterns.

#### 4.2.2.1 The rotating snake

**Figure 6:**
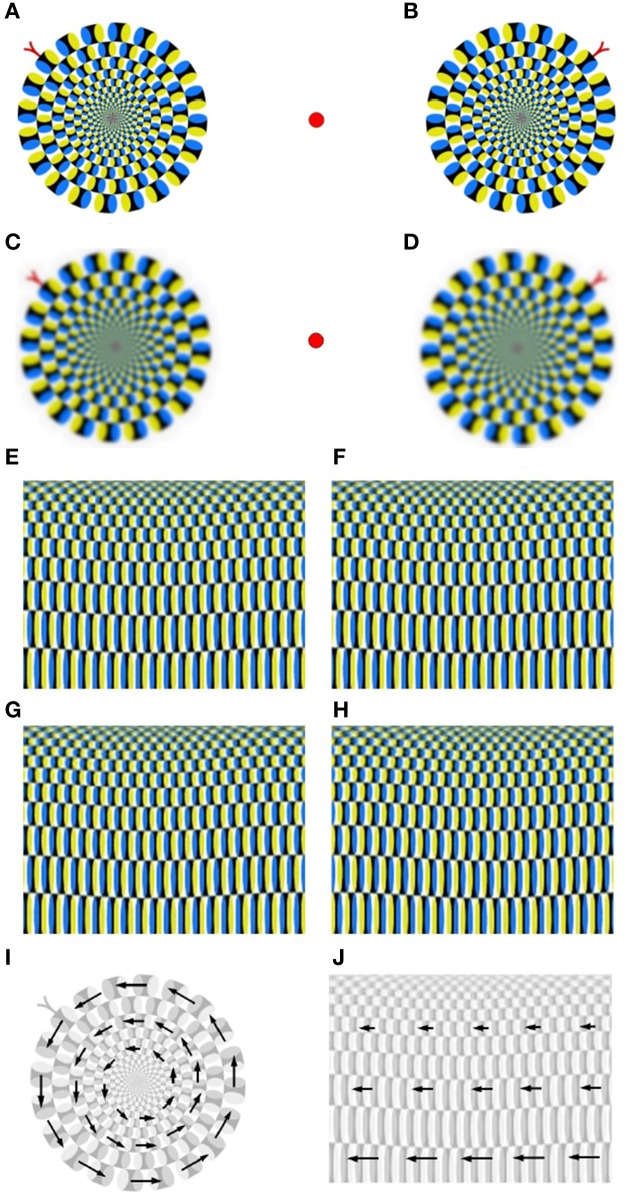
**Apparent motion illusions. (A,B)** A pair of the Rotating Snake patterns from Kitaoka and Ashida (2003). **(C,D)** The low-pass filtered Rotating Snakes exhibit slower rotation. **(E,F)** Parallel patterns of the polar-to-rectangle transformation of the Rotating Snake, i.e., the Translating Snake, does not produce any motion illusions. **(G,H)** Antiparallel patterns of the Translating Snake generates a weak illusion of apparent translational motion. **(I,J)** Visualization of the Rotating and Translating Snake pattern shows that motion elements of the Rotating Snake exhibit a relative phase-shift to each other, while the Translating Snake elements are parallel and do not have any relative phase shift.

patterns from Kitaoka and Ashida (2003) induce a remarkably strong illusion of apparent rotational motion, see Figures 6A,B. The low-pass smoothed Rotating Snake in Figures 6C,D exhibit a reduced intensity of apparent rotational motion. Backus and Oru (2005) explain emergence of illusory motion of the Rotating Snakes by the difference in the temporal response of visual neurons to low- and high-contrast. This difference leads to misinterpretation of the temporal phase-shift as a spatial phase-shift (“phase advance”) at high contrast. The effect of low-pass smoothing, authors attribute to reduction of differences between high- and low-contrast regions. Recent findings indicate that signals of illusory motion in V1 and MT cortical areas can be also triggered by update of the retinal image as a result of saccadic eye movements or blinkers (Conway et al., 2005; Troncoso et al., 2008; Otero-Millan et al., 2012; Martinez-Conde et al., 2013). Consequently, conscious suppression of saccades inhibits illusions of apparent motion that are based on phase-advancing contrast patterns. To dissect the structural principle of the Rotating Snake in more detail, we performed its polar-to-rectangle transformation into the Translating Snake, see Figures 6E–H. This transformation changes the relative spatial orientation of repetitive patterns while preserving their local contrast structure. We observe that a pair of parallel Translating Snake patterns does not induce any significant perceptual effects, see Figures 6E,F. In contrast, antiparallel Translating Snakes patterns generate a weak illusion of translational motion, see Figures 6G,H. From this observation, we conclude that phase advancement due local contrast gradient is required but not sufficient for generation of apparent motion illusion. The sufficient condition consists in different spatial orientation of repetitive motion patterns: equally oriented motion patterns of the Translating Snake do not induce any illusory motion, while non-uniformly organized contrast gradients of the Rotating Snake do, see Figures 6I,J. Thus, we conclude that apparent motion signals are triggered not only by phase advancement at high contrast alone but by the difference in phase advancement between each two image regions subsequently fixated by saccades.

#### 4.2.2.2 The anomalous motion

**Figure 7:**
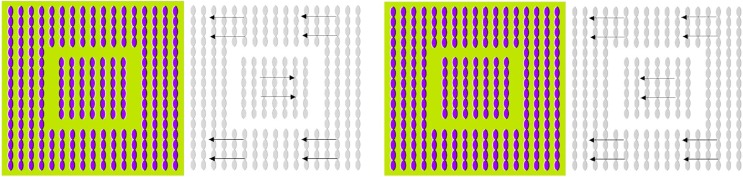
**The Anomalous Motion (courtesy A. Kitaoka) induces an illusion of apparent translational motion (left)**. Manipulated equidirectional stimulus **(right)** do not trigger any significant motion illusions.

from Kitaoka (2006) is another example of apparent motion illusion which is induced by contrarily oriented contrast-gradient patterns, see Figure 7 (left). In Figure 7 (right), central and peripheral contrast-gradient patterns were aligned in the same direction. As a result, the illusion of apparent motion disappears. Only the combination of patterns with contrarily oriented contrast-gradients (i.e., the relative phase shift) is capable to generate a stable illusion of apparent relative motion, see Figure 7 (left). Similar to the Rotation Snake, the Anomalous Motion illusion requires saccadic eye movements. Suppression of saccades by conscious point fixation stops the illusion of apparent motion.

#### 4.2.3. Non-local tilt illusion.

**Figure 8:**
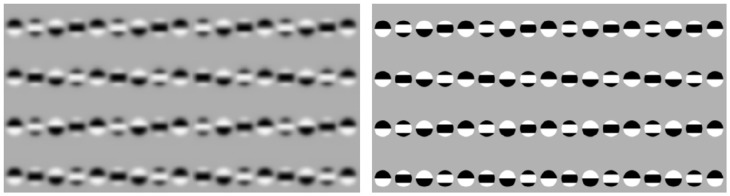
**Dependence of the non-local tilt illusion on low/high-frequent image content**. From left to right: the low-pass filtered vs. unfiltered Popple illusion (courtesy A. Kitaoka).

Figure 8 shows the virtual tilt illusion from Popple and Levi (2000) and Popple and Sagi (2000) which seems to be triggered without local cues. The particularity of this stimulus consists in a way it is constructed by horizontal lines of patterns that exhibit a relative vertical phase-shift. Consequently, the horizontal lines appear to have a vertical tilt which direction depends on the sign of the phase-shift. Based on our previous analysis of motion illusions, we presume that also the virtual tilt illusion is driven by saccadic eye motions along the horizontal lines of patterns. Consequently, the virtual tilt illusion is, nevertheless, based on local cues that are established by successive saccadic fixations.

Another puzzling property of this stimulus is the dependency of the tilt intensity on spectral image characteristics. Remarkably, the low-pass smoothed stimulus seems to exhibit stronger tilt as the unsmoothed version with high-frequent components. One possible explanation for this observation is that phase correlation of low-pass smoothed patterns results in a wide and blurry shift signal, cf. Figure 3. Another hypothetic assumption is that the strategy of saccadic eye movements differs for low-pass smoothed and unsmoothed stimuli. If, for instance, saccadic sampling of blurry images turns out to be associated with faster and/or more distant jumps,—this can effectively lead to stronger shift perception in comparison to unsmoothed stimuli.

## 5. Pattern recognition using phase correlation

As we have seen above, pattern recognition and motion detection are closely related tasks in the frequency domain. In fact, detection of pattern motion using phase correlation premises the knowledge of complete spectral characteristics of a pattern, i.e., pattern recognition. The tight relationship between pattern's cognitive characteristics and motion can be seen as an exclusive feature of frequency domain techniques such as phase correlation, which differs them, for example, from gradient-based optical flow methods (Barron et al., 1994). The existing body of neurophysiological and psychophysical evidence do not allow to make a conclusion about the nature of neural mechanisms of pattern recognition. However, from the literature it is known that (i) the retinal images are frequency-coded, filtered and processed in visual cortex by several layers of specialized cells in a hierarchically organized manner (Mesulam, 1998; Kruger et al., 2013), (ii) recognition takes place in higher levels of this hierarchy, i.e., the association cortex, where high confidence pattern recognition has been related to activity of single cells (Quiroga et al., 2005), and (iii) saccades are involved in acquisition of the information for rapid scene recognition (Kirchner and Thorpe, 2006). By putting these findings together with our theoretical and experimental investigations, we hypothesize here that phase correlation (or an effectively similar mechanism) is involved in neural machinery of pattern recognition. The basic statements of this hypothesis are as follows:

- Images are coded in the neural network by their frequency domain features (i.e., phases and amplitudes).
- Phase correlation between neural images is performed by a special layer of cells [further termed as association layer neurons (ALN)].
- Similarity between each two visual stimuli is sensed by the spatial-temporal pattern of ALN activity in analogy to *PC* of two images, cf. Figure 3.

**Figure 9:**
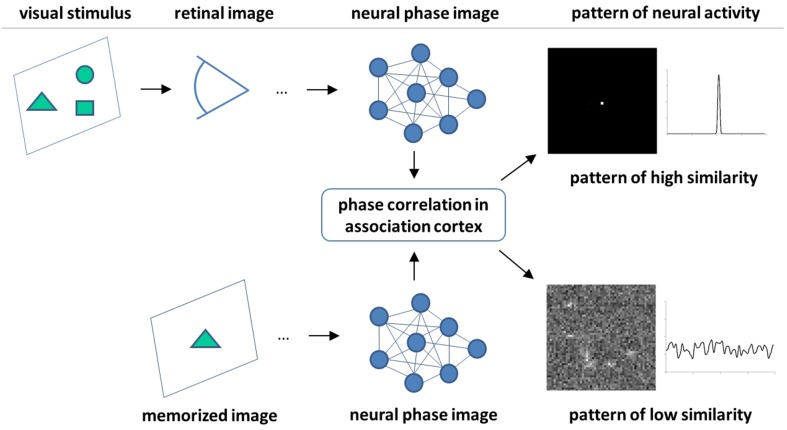
**Scheme of the hypothetic mechanisms of visual pattern recognition**. Persistent activity of a small number of neurons in association cortex is a feature of high image similarity. In the ideal case, similarity is detected by a single neuron. In contrast, a more disperse and stochastic pattern of neural activity indicates a low degree of image similarity.

Figure 9 depicts the principle scheme of this hypothetic mechanism which postulates integration (phase correlation) of source and target images in association cortex and predicts the neural activity patterns related to perception of image (dis)similarity. According to this hypothesis, the physiological expression of high-confidence recognition of a visual stimulus is a coherent and persistent activity of a relatively small number of ALN (theoretically, even one single neuron as it has been observed in Quiroga et al. (2005)). In contrast, low similarity between visual stimuli would result in a diffuse and uncorrelated pattern of ALN activity. Furthermore, missing similarity between images can be expected to provoke intensification of saccadic eye movements.

**Figure 10:**
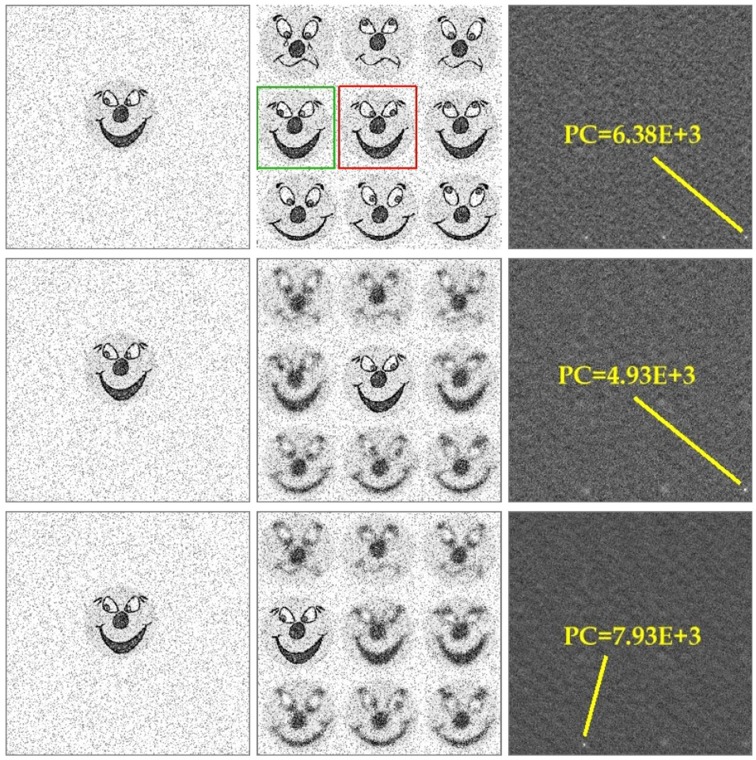
**Example of pattern recognition using phase correlation**. From left to right: (i) the target smiley, (ii) multi-smiley image, phase correlation between (i) and (ii). The green frame indicates the correct location of the target pattern in the image, the red frame shows the wrong match which corresponds to the absolute maximum of the noisy phase correlation. Consideration of visual acuity improves the recognition score. Phase correlation between the target smiley and the images with three different acuity foci peaks out the right pattern location with the maximum height of PC = 7.93E + 3.

An example of repetitive pattern discrimination/recognition using phase correlation is shown in Figure 10. The task consists in finding a particular smiley within a group of similar patterns. Since phase correlation of noise-free images will immediately match the right location of the target smiley, the search is complicated by adding a large amount of high-frequency noise which substantially corrupts small image features (such as smiley's eyes). Single-step phase correlation between substantially noised images results in selection of the wrong pattern location (see yellow framed smiley in Figure 10). Due to high-level of noise, the peak of phase correlation corresponding to the correct pattern (green framed smiley) has the lower height. Remarkably, consideration of visual acuity (i.e., peripheral blurring) helps to improve the recognition score. Phase correlation between the target smiley and three images with different visual foci manages to peak out the right pattern location which corresponds to the highest peak of *PC* = 7.93*E* + 3.

**Figure 11:**
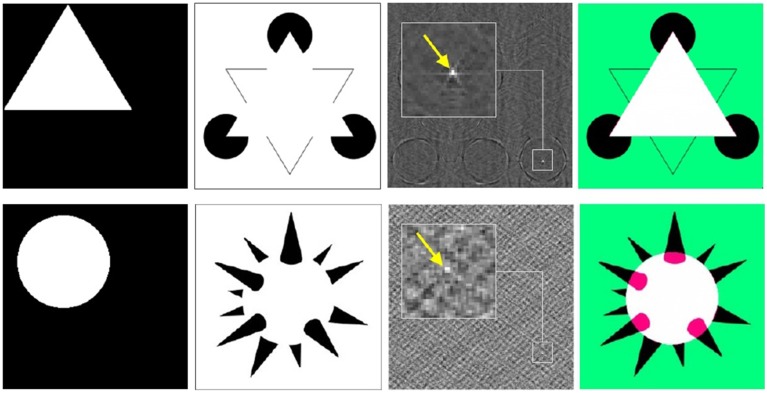
**Detection of the virtual image content using phase correlation**. From left to right: (i) hidden patterns of illusion stimuli (i.e., triangle, circle), (ii) visual completion illusion from Kanizsa (1995) (top row) and Idesawa (1991) (bottom row), (iii) phase correlations between (i) and (ii) (maximum is indicated by the arrow), registration of (i) onto (ii) according to the maximum of (iii).

Another example of remarkable features of phase correlation as a pattern recognition tool is detection of the virtual image content in visual completion illusions. Figure 11 demonstrates detection of virtual geometrical patterns (i.e., triangle, circle) in the completion illusions from Idesawa (1991) and Kanizsa (1995). The correct location of the virtual figures corresponds to the absolute maximum of phase correlation. This examples demonstrate that phase correlation is capable to retrieve even extremely subtle pattern correspondences.

## 6. Discussion

Here, we merge existing phenomenological findings, computational analysis and theoretical hypotheses to dissect the role of image phase in diverse phenomena of visual information processing, illusion and cognition. We argue that fundamental importance of phase for detection of structural image features and transformations is of clear evolutionary advantage for survival of species and can be assumed to promote the development of phase-based mechanisms of neural image processing. A large body of neurophysiological and psychophysical evidence seems to confirm the assumption that biological vision relies on frequency domain transformation, filtering and higher-order processing of retinal images in the visual cortex. Hence, the emergence of efficient phase-based neural mechanisms in course of evolution appears to be plausible. We show that the concepts of phase shift, amplitude-normalizing phase-only transformation and phase correlation provide a qualitative description for a number of puzzling visual phenomena including

- preservation of cognitive features in the image sketch (in the sense of the Marr's Primal Sketch),
- robustness of pattern detection with respect to substantial level of noise and structural distortion,
- “eye exhaustion” by observation of repetitive and blurry scenes,
- advantages of saccadic strategy of iterative target-background sampling for pattern discrimination,
- dependency of saccadic eye movements on structural image properties (i.e., target-background similarity and spectral characteristics),
- advantages of differences in foveal and peripheral acuity for visual pattern recognition,
- dependency of the delay time by perception of virtual depth illusions on phase properties of stimuli,
- coherent phase shifts in contrast-gradient patterns of apparent motion illusions,
- driving role of saccades in apparent motion and tilt illusions,
- recognition of virtual patterns in completion illusions using phase correlation.
- singular pattern of neural activity in the association cortex by recognition of similar visual stimuli.

Although, straightforward projections of theoretical concepts onto biological systems can, in general, lead to too far-reaching extrapolations, some of our hypothetic predictions, such as dependency of saccades strategy on structural image properties and singular response of association cortex to structurally similar visual stimuli, can be, on principle, tested in experiment.

There is a tight resemblance between the concepts of amplitude-normalizing phase-only transformation and phase correlation we used in our work and energy models (Morrone and Owens, 1987; Morrone and Burr, 1988; Fleet et al., 1996) re. phase congruency detectors (Morrone et al., 1986; Kovesi, 2000). Both concepts take advantage of two basic principles: (i) amplitude-normalization, which effectively performs edge enhancement (i.e., image sketchification) and makes scene analysis independent of the level of illuminance and contrast, and (ii) calculation of the cognitive checksum by building an integral over the entire frequency spectrum, which, on one hand, makes the cognition extremely robust with respect to noise and, on the other hand, allows distributed storage of information in neural networks. Otherwise, there is a basic difference between these two concepts: phase congruency can be seen as an extended amplitude-normalizing, edge-enhancing filter, while phase correlation is constructed to detect the relative transformation and/or structural (dis)similarity between each two images. Furthermore, phase congruency is presumably performed by V1 neurons, while phase correlation can be expected to take place in a higher level of visual cortex hierarchy, i.e., association cortex. Finally, taking into consideration potential redeployment of the brain areas (Anderson, 2007), one can expect that the suggested principle of pattern recognition by phase correlation is not restricted to the visual system and could also play a role in other cognitive functions.

Within the general framework of recent hierarchical bottom-up top-down models of visual cortex (Lee and Mumford, 2003; Epshtein et al., 2008; Poggio and Ullman, 2013), our findings provide a theoretical explanation for what Marr called “early non-attentive vision” (Marr, 1976, 1982). In particular, our above results suggest that phase-only transformation in V1 with subsequent phase correlation in association cortex represent bottom-up neural mechanisms of Primal Sketch generation and perception, respectively. However, differently from the canonical edge operators that are based on derivatives (i.e., edge-mask convolution) of the image intensity function, edge information in the frequency domain is given implicitly by the relative phase structure and can be assessed for the entire image in a non-iterative and non-local manner. The ability of phase correlation to capture global structural information “on-the-fly” makes it to an ultimate tool for rapid bottom-up processing of the focused image content. The temporal focus of the observer is, in turn, controlled by higher-order cortical centers that integrate bottom-up streams and define conscious and unconscious strategies of visual scene sampling.

While the focus of our present work is on the role of image phase in visual information processing, it should be stated that phase does not exclusively bear cognitive features of visual stimuli. Findings in Freeman and Simoncelli (2011) and Zhang et al. (2014) suggest that amplitude information is also involved in visual (re)cognition and can be even overweight in peripheral vision or by perception of textural images. It is a subject of future research to reveal how phase and amplitude are weighted and merged to an integrated whole in association cortex upon structural properties of visual stimuli.

### Conflict of interest statement

The authors declare that the research was conducted in the absence of any commercial or financial relationships that could be construed as a potential conflict of interest.

## Footnotes

^1^Reformulation of phase correlation in polar coordinates results in detection of the image scaling and rotation (Reddy and Chatterji, 1996).

^2^All examples of visual stimuli were taken from the “Illusion Pages” of A. Kitaoka http://www.psy.ritsumei.ac.jp/akitaoka/cataloge.html.
